# Treatment outcome and associated factors of severe acute malnutrition among 6–59 months old children in Debre Markos and Finote Selam hospitals, Northwest Ethiopia: a retrospective cohort study

**DOI:** 10.1186/s40795-017-0161-3

**Published:** 2017-05-15

**Authors:** Getnet Mekuria, Tariku Derese, Getachew Hailu

**Affiliations:** 1 Department of Applied Human Nutrition, Bahir Dar Institute of Technology, Bahir Dar University, Bahir Dar, Ethiopia; 2 Finote Selam District Hospital, Finote Selam, Ethiopia; 3 Department of Public Health, College of Health Sciences, Debre Markos University, Debre Markos, Ethiopia

**Keywords:** Folic Acid, Folic Acid Supplementation, Severe Acute Malnutrition, Sphere Project, Complementary Feeding Practice

## Abstract

**Background:**

In Ethiopia, the health sector has increased its efforts to enhance good nutritional practices through health education, treatment of extremely malnourished children and provision of micronutrients for mothers and children. But, the poor nutritional status of women and children continues to be still a major public health problem.

**Methods:**

A retrospective cohort study was conducted to assess the treatment outcome and associated factors of severe acute malnutrition among a total of 253 children age 6–59 months old. Severe acute malnutrition registration logbook and patient charts were used as a source of data. Data were entered in to Epi-data version 3.1 and exported to SPSS version 20 for analysis. To identify associated factors, Cox proportional hazard analysis was computed and *p*-value <0.05 at 95% confidence interval was considered as statistically significant.

**Results:**

The recovery rate was 77.9% and the overall median recovery time was 11 days. Those children age from 24 to 35 months had 34% lower probability of recovery from SAM compared to 6–11 months old children (AHR = 0.66, 95% CI: 0.35–0.89). Children whose ages from 36 to 59 months had 47% lower probability of recovery from SAM compared to 6–11 months old children (AHR = 0.53, 95% CI: 0.31–0.91). HIV negative children had 2.48 times higher probability of getting recovered from SAM compared to HIV positive children (AHR = 2.48, 95% CI: 1.23–5.01). Children who didn’t take folic acid supplement had 65% lower probability of recovery from SAM compared to children who took folic acid supplement (AHR = 0.35, 95% CI: 0.14–0.89).

**Conclusions:**

This study found that recovery rate of 6–59 months old children treated for severe acute malnutrition in therapeutics units was in acceptable range based on the WHO recommendation. Folic acid supplementation and screening for HIV status should be promoted at all levels of health facilities during early age.

## Background

Annually, severe acute malnutrition (SAM) is responsible for the death of 3.6 million under 5 years of old children and 140.5 million Disability Adjusted Life Years (DALYs) of children in the same age groups. When we consider from economic perspective, there is a need to break the cycle of malnutrition and poverty as estimates suggest that failing to address them results in a 2.3% loss in national gross domestic product (GDP) [1].

Severe acute malnutrition affects nearly 20 million pre-school age children, mostly in Sub-Saharan African and South East Asia region. Worldwide, malnutrition is a significant factor in approximately one third of the nearly 8 million deaths in under 5 years of old children [2].

Malnutrition has a dramatic impact on childhood mortality still in Sub-Saharan African countries including Ethiopia [3]. Previous studies conducted in Ethiopia from Mekele city of Tigray [4] and University of Gondar Hospital [5] found that unacceptable high case fatality rate of 12.8 and 18.4% respectively.

In Ethiopia, the health sector has increased its efforts to enhance good nutritional practices through health education, treatment of extremely malnourished children and provision of micronutrients for mothers and children. But, the poor nutritional status of women and children continues to be a series problem still. An estimated 312,211 children required treatment for SAM in 2012G.C.The near complete failure of the 2012 February–May rains resulted in increased food insecurity and malnutrition in Southern Nations, Nationalities, and Peoples’ Region, part of Amhara and Oromiya Regions [6].

Ethiopia Demographic and Health Survey (EDHS) 2016 report showed that 38, 10 and 24% of under 5 years of age children in Ethiopia were stunted, wasted and underweight respectively. In the Amhara region, 46% of under 5 years of age children were stunted which was the highest in the country [7].

Even though malnutrition is one of the public health problems in Ethiopia, limited information is available on inpatient treatment outcome of SAM and associated factors in Debre Markos and Finote Selam Hospitals. In general, evaluating and auditing the treatment outcome and mortality rates of malnourished children at therapeutic centers are very useful for countries to identify the gaps and measures the effectiveness of center based management of severe acute malnutrition for future to develop best interventional approach. Therefore, the aim of this study was to assess treatment outcome and associated factors of SAM recovery among 6–59 months old children in Debre Markos and Finote Selam Hospitals.

## Methods

### Study setting and participants

Institution based retrospective cohort study was conducted from April 20/2016 to April 24/2016 in Debre Markos Referral and Finote Selam District Hospitals, located 295KM and 387KM Northwest of Addis Ababa, Ethiopia. Approximately, 3.5 million and 100,000 people were served by these two hospitals respectively. The total numbers of children served in their catchment areas were 450,800. The study populations were severely malnourished children age 6–59 months old admitted with SAM in therapeutic feeding centers of Debre Markos Referral and Finote Selam District Hospitals from April 2014 to April 2016.

Study participants consist of all 253 eligible (out of total 321 severely malnourished children age 6–59 months old admitted in therapeutic feeding centers of Debre Markos Referral and Finote Selam District hospitals) from April 2014 to April 2016. A total of 2 year records of SAM inpatient cases in the two hospitals were 321 cases and categorized separately from those in Debre Markos Referral hospital (182) cases and those records in Finote selam hospital (139) cases. But only 139 cases from Debre Markos Referral hospital and 114 cases from Finote selam hospital had complete data charts for this study. Finally 253 severe acute malnutrition cases from both hospitals were taken as study participants.

Admission criteria for 6 months to 18 years old children in the therapeutic units were as follows: WFH < 70% or WFH less than −3 Z-score or WFL < 70% or WFL less than −3 Z-score or MUAC <110 mm with length > 65 cm or presence of bilateral pitting edema/complication. The discharge criteria for 6 months to 18 years old children in the therapeutic units were as follows: W/L > =85% or W/H > =85% on more than one occasion (2 days for in-patients, 2 weeks for out-patients) and no oedema for 10 days (In-patient) or 14 days (out-patient) [8].

### Inclusion criteria

WFH < 70% or WFH less than −3 Z-score or WFL < 70% or WFL less than −3 Z-score or MUAC <110 mm with length > 65 cm or presence of bilateral pitting edema/complication treated from January 2014 to January 2016 in therapeutics feeding centers of selected hospitals.

### Exclusion criteria

SAM Children treated at outpatient therapeutic units or inpatient SAM cases who had in complete data charts.

### Operational definitions

Recovery is reaching >80% of nutritional median WFH and in patient treatment outcome logbook declared as improved or recovered [8].

Defaulters- are those patients who leaves from treatment unit against medical advice and declared as defaulter or against in the treatment logbook [8].

Death- refers to the patient that has die while he/she is in the therapeutic units and declared as death in the treatment logbook [8].

$$ \mathrm{Recovery}\ \mathrm{rate}=\mathrm{No}\ \mathrm{of}\ \mathrm{patient}\ \mathrm{discharged}\ \mathrm{for}\ \mathrm{recovery}/\mathrm{Total}\ \mathrm{No}\ \mathrm{of}\ \mathrm{exits} $$

$$ \mathrm{Defaulter}\ \mathrm{rate}=\mathrm{No}\ \mathrm{of}\ \mathrm{true}\ \mathrm{defaulters}/\mathrm{Total}\ \mathrm{No}\ \mathrm{of}\ \mathrm{exits} $$

$$ \mathrm{Death}\ \mathrm{rate}=\mathrm{No}\ \mathrm{of}\ \mathrm{patient}\ \mathrm{died}\ \mathrm{in}\ \mathrm{the}\ \mathrm{programme}/\mathrm{Total}\ \mathrm{No}\ \mathrm{of}\ \mathrm{exits} $$

$$ \mathrm{Average}\ \mathrm{length}\ \mathrm{of}\ \mathrm{stay}=\mathrm{Sum}\;\mathrm{of}\ \mathrm{length}\ \mathrm{of}\ \mathrm{stay}/\mathrm{No}\ \mathrm{of}\;6\hbox{-} 59\;\mathrm{months}\ \mathrm{cured} $$

Censor = refers to defaulter from treatment, transfer out, those who died with indirect and direct causes and those cases not known the result at the end of the study period.

The event (outcome of interest) = was consider to be recovery of severely malnourished children while in the therapeutic units**.**

### Data collection

Data were collected from SAM registration Logbook and medical record charts by using checklist. Data collection checklist cross checked with pre-established known source [3] to address the study variables. Data were collected by nurses who took training on the management of SAM. Data collectors were trained for 1 day and daily supervised by investigators.

### Data processing and analysis

Data were entered into Epi data version 3.1 and analysis was done by using SPSS Version 20 software. Kaplan-Meier and Cox regression were used to assess the association of independent variables with time to recovery. First bivariate Cox regression analysis computed for each predictor variable with time to recovery. Then variables associated with time to recovery at 0.2 significant levels were included in the multivariate Cox proportional Hazard model. Hazard ratio (HR) with 95% CI was used to identify predictor variables. Variables which had *p*-value < 0.05 were considered as significant.

## Results

### Socio-demographic characteristics

More than half (54.8%) of the children enrolled into the study were females and 39.9% were in the age group of 6–11 months with median age of 13 months. The majority (83.8%) of severely malnourished children were from rural area (Table 1).

**Table 1:** Socio-demographic characteristics of children 6–59 month old in the therapeutic units of Debre Markos Referral and Finote Selam District Hospitals, 2016

Characteristics		Frequency (*n* = 253)	Percent
Age group (Months)	6–11	101	39.9
	12–23	94	37.2
	24–35	32	12.6
	> = 36	26	10.3
Sex	Male	115	45.2
	Female	138	54.8
Residence	Urban	41	16.2
	Rural	212	83.8

### Medication provision, major co-infection, and vitamin supplementation in the therapeutics centers

Among admitted children 64.9% of them had co-infection and the most common co-infections were diarrhea (28.2%), pneumonia (23.3%), anemia (18.4%) and tuberculosis (11.7%). The most commonly prescribed drugs were amoxaciline (28.1%) and ampiciline (16.6%). In the therapeutics centers, 82.6% and 83.8% of admitted children with SAM received vitamin A and folic acid supplementations respectively (Table 2).

**Table 2:** Medication provision, major co-infection and mineral supplementation in the therapeutics centers of Debre Markos Referral and Finote Selam District Hospitals, 2016

Characteristics	Frequency (*n* = 253)	Percent
Vitamin A supplemented		
Yes	209	82.6
No	44	17.4
Folic acid supplemented		
Yes	212	83.8
No	41	16.2
Medications provided		
Amoxaciline	71	28.1
Ceftriaxone	25	9.9
Cephalexin	31	12.3
Ampiciline	42	16.6
Penciline	9	3.6
Gentamicine	35	13.8
cotrimoxazole	34	13.4
Others	6	2.4
Co-infection		
Yes	163	64.4
No	90	35.6
Major co-infection (*n* = 163)		
Diarrheoa	46	28.2
Pneumonia	38	23.3
Anemia	30	18.4
Dehydration	10	6.1
Fever	12	7.4
TB	19	11.7
Others	8	4.9

### Treatment outcome of SAM by therapeutics food provision in the treatment centers

**Fig. 1:**
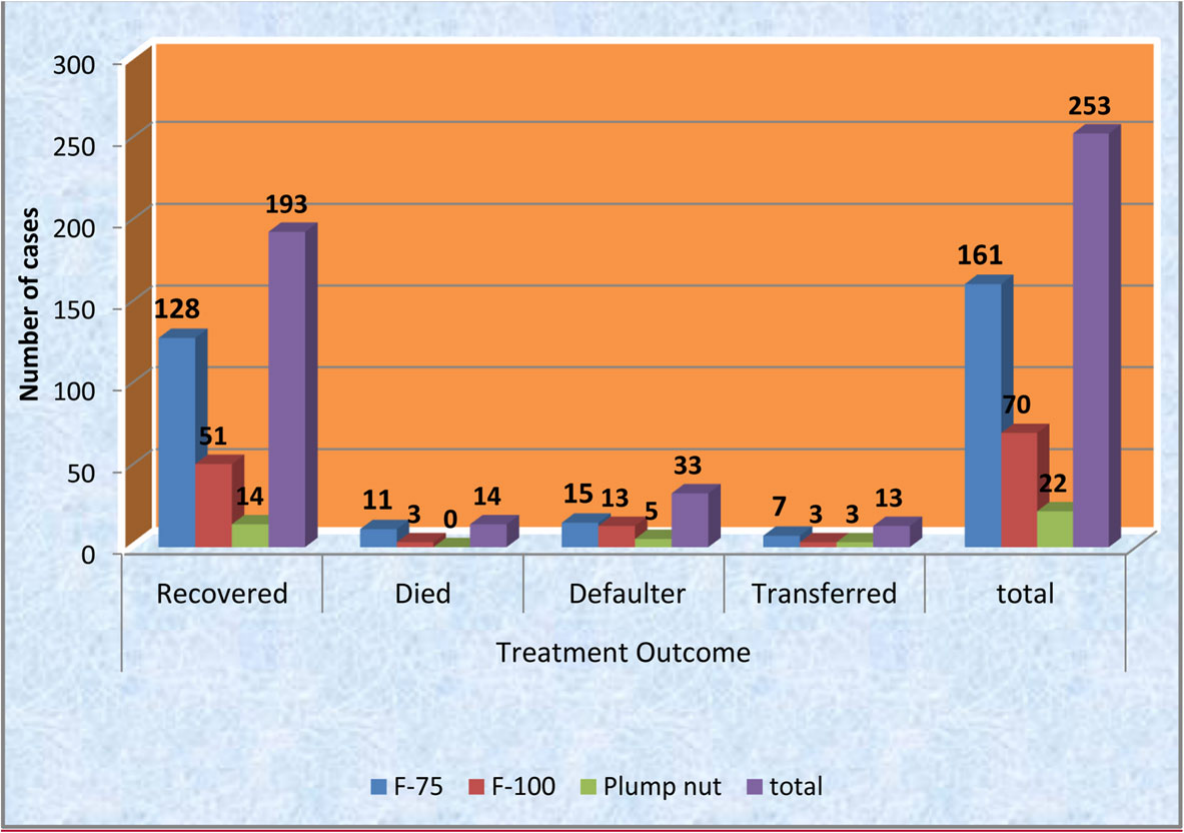
Treatment outcome of SAM by provision of therapeutic foods in the therapeutic units of Debre Markos Referral and Finote Selam District Hospitals, 2016

The most commonly used therapeutics products in the management process were F-75 (64.5%) and followed by F-100(27.4%). Among children who were taking f-75, 79.5%(128) were recovered and 6.8%(11) were died, but among who were taking the f-100 the recovery was 72.8%(51) and death were 4.2%(3) (Fig. 1).

### Treatment outcome of SAM by type of diagnosis in the treatment centers

The predominant (75.5%) form of malnutrition in this study was marasmus. Among children diagnosed as kwashiorkor, 74.5%(38) were recovered, death rate 5.9% and defaulter rate was 13.7% [7] (Table 3).

**Table 3:** Treatment outcome of SAM by type of diagnosis in the therapeutic centers of Debre Markos Referral and Finote Selam District Hospitals, 2016

		Treatment Outcome				
		Recovered *n* (%)	Died *n* (%)	Defaulter *n* (%)	Transfer *n* (%)	Total *n* (%)
Diagnosis	Kwashiorkor	38 (74.5%)	3(5.9%	7(13.7%)	3(5.9%)	51(100.0%)
	Marasmus	148(77.5%	11(5.8%)	24(12.6%)	8(4.2%)	191(100.0%)
	kwashiorkor-marasmus	11(100.0%)	0(.0%)	0(0%)	0(0%)	11(100.0%)
Total		197(77.9%)	14(5.5%)	31(12.3%)	11(4.3%)	253(100.0%)

### Kaplan-Meir survival estimates for sever acute malnutrition recovery time and type of health facility

The median survival time of recovery for children admitted in Debre Markos Referral Hospital was 11 days with 95%CI (10.061–11.939) and in Finote Selam District Hospital; it was 11 days with 95%CI (9.849–12.151). The overall median survival time for this study was 11 days with 95%CI (10.471–11.529) (Table 4).

**Table 4:** Kaplan-Meir survival estimates for sever acute malnutrition recovery time with type of health facility at the therapeutics centers Debre Markos Referral and Finote Selam District Hospitals, 2016

Type of Hospital	Mean	Std. Error	95% Confidence Interval		Median	Std. Error	95% Confidence Interval	
Referral	12.487	.729	11.059	13.915	11.000	.479	10.061	11.939
District	13.922	.871	12.216	15.629	11.000	.587	9.849	12.151
Overall	13.150	.562	12.047	14.252	11.000	.270	10.471	11.529

### Hazard function of type of health facility with recovery time

**Fig. 2:**
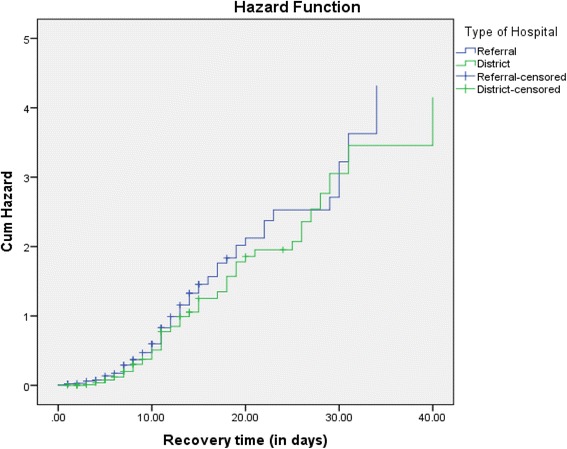
Log rank survival estimates for severely malnourished children with type of health facility with time to recovery in the therapeutic units of Debre Markos Referral and Finote Selam District Hospitals, 2016

The log rank survival curves of severely malnourished children admitted in the referral and district hospitals cross each other showed that there was no significant difference of hazard risk between severely malnourished children admitted in the two hospitals (Fig. 2).

### Treatment outcome compared to sphere project value/international standard of severe acute malnutrition in treatment centers

Out of 253 children whose records were reviewed, 197 (77.9%) were recovered, 14(5.5%) died during treatment, 31(12.3%) defaulted and 11(4.3%) transferred from treatment centers. The average length of stay in the hospitals was 11.1 days (Table 5). These results of treatment outcome of severe acute malnutrition in therapeutics units were in acceptable range compared to the SPHERE project reference values [9].

**Table 5:** Performance indicator values of inpatient therapeutic feeding centers of Debre Markos Referral and Finote Selam District Hospitals as compared to sphere project reference values/international standard, 2016

Performance indicators	Finote Selam	Debre Markos Referral	The SPHERE project reference values		
	Hospital	Hospital	Over all	Acceptable	Alarming
Recovery rate	77.2%	78.4%	77.9%	>75%	<50%
Death rate	6.1%	5%	5.5%	<10%	>15%
Defaulter rate	12.3%	12.2%	12.3%	<15%	>25%
Average length of stay	11.7 days	10.6 days	11.1 days	<28 days	>42 days
Transfer rate	4.4%	4.3%	4.3%		

### Factors associated with recovery time of severely malnourished children

During the bivariate Cox regression analysis; age group, place of residence, HIV status, presence of co-infection, type of diagnosis and folic acid supplementation were significantly associated with recovery time of SAM (Table 6). However during the multivariate Cox regression analysis; age group, HIV status and folic acid supplementation were significantly associated with recovery time of SAM (Table 7).

**Table 6:** Bivariate analysis (Cox regression) of factors associated with recovery time of SAM among severely malnourished in the therapeutic units of Debre Markos Referral and Finote Selam District Hospitals, 2016

Factors (variables)	No	CHR	95%CI	*P*-value
Age group (in months)				
6–11	101	1		
12–23	94	0.74	0.54–1.02	0.07
24–35	32	0.64	0.40–1.01	0.06
36–59	26	0.55	0.32–0.92	0.02
Residence				
Urban	41	1		
Rural	212	0.67	0.47–0.97	0.04
HIV status				
Positive	18	1		
Negative	235	2.98	1.003–8.87	0.03
Co-infection				
Yes	163	1		
No	90	1.25	0.93–1.67	0.14
Type of diagnosis				
Kwashiorkor	51	1		
Marasmus	191	0.97	0.68–1.39	0.86
Marasmic- Kwashiorkor	11	2.49	1.26–4.93	0.01
Folic acid supplementation				
Yes	212	1		
No	41	0.41	0.24–0.92	0.08

**Table 7:** Multivariate analysis (Cox regression) of factors associated with recovery time of SAM among severely malnourished in the therapeutic units of Debre Markos Referral and Finote Selam District Hospitals, 2016

Factors (variables)	CHR	95% CI	AHR	95% CI	*P*-value
Age group (in months)					
6–11	1		1		
12–23	0.74	0.54–1.02	0.73	0.52–1.01	0.06
24–35	0.64	0.42–1.01	0.66	0.35–0.89	0.02
36–59	0.55	0.32–0.92	0.53	0.31–0.91	0.02
HIV status					
Positive	1		1		
Negative	2.98	1.003–8.87	2.48	1.23–5.01	0.01
Folic acid supplementation					
Yes	1		1		
No	0.41	0.24–0.92	0.35	0.14–0.89	0.03

Those children age from 24 to 35 months had 34% lower probability of recovery from SAM compared to 6–11 months old children (AHR = 0.66, 95% CI: 0.35–0.89). Children whose ages from 36 to 59 months had 47% lower probability of recovery from SAM compared to 6–11 months old children (AHR = 0.53, 95% CI: 0.31–0.91). HIV negative children had 2.48 times higher probability of getting recovered from SAM compared to HIV positive children (AHR = 2.48, 95% CI: 1.23–5.01). Children who didn’t take folic acid supplement had 65% lower probability of recovery from SAM compared to children who took folic acid supplement (AHR = 0.35, 95% CI: 0.14–0.89).

## Discussion

This study analyzed the treatment outcomes of infants and children 6–59 months age who have MUAC <11.5 cm or bilateral pitting edema and co-infection. Findings of this study showed that the recovery rates and death rates among admitted 6–59 months old children were 77.9 and 5.5% respectively. Not only recovery and death rate but also the other outcome indicators in this study showed that there were in the minimum standard set of sphere project values/international standards [9].

The recovery rate in our study is higher than previous findings from Tigray [10], Kamba District [11], Uganda [12], Sudan [13], Tamale Teaching Hospital [14] and India [15]. But it is lower than findings from Jimma University Specialized Hospital [16], Woldiya General Hospital [17], Southern region of Ethiopia [18] and Rural Ethiopia [19]. This difference could be due to differences in socio-economic status, quality of care provided for children, health seeking behavior, availability as well as accessibility of therapeutic foods and medications. Another possible factor for this variation could be guideline up date for SAM treatment.

The present study also found that higher mortality rate than reports from Tigray [10], Southern region of Ethiopia [18] and India [15]. However, it is lower than reports from Uganda [12], Sudan [13], Jimma University Specialized Hospital [16], Woldiya General Hospital [17] and Rural Ethiopia [19]. The possible explanation for this variation might be differences in quality of services provided for children admitted with SAM and management of medical complications associated with SAM.

The presence of HIV infection among children was negatively associated with recovery time from SAM. HIV negative children had 2.48 times higher probability of getting recovered from SAM compared to HIV positive children (AHR = 2.48, 95% CI: 1.23–5.01). This is in line with Woldiya General Hospital [17] finding. It is known that the effects of HIV/AIDS and malnutrition are interconnected and worsen one another in a vicious cycle.

Being in the younger age group was positively associated with recovery time from SAM. Those children age from 24 to 35 months had 34% lower probability of recovery from SAM compared to 6–11 months old children (AHR = 0.66, 95% CI: 0.35–0.89). Children whose ages from 36 to 59 months had 47% lower probability of recovery from SAM compared to 6–11 months old children (AHR = 0.53, 95% CI: 0.31–0.91). This finding is similar with previous results [11, 16]. This might be due to discontinuation of breastfeeding and inappropriate complementary feeding practices as children’s age increases.

Folic acid supplementation was positively associated with recovery time from SAM. Children who didn’t take folic acid supplement had 65% lower probability of recovery from SAM compared to children who took folic acid supplement (AHR = 0.35, 95% CI: 0.14–0.89). This could be due to the fact that folic acid supplementation prevents anemia.

The limitations of this study were lack of comparison group from other healthcare facilities in the region, lack of information about whether there were cases of relapse—cases being readmitted shortly after discharge, possible reasons being use of inappropriate discharge criteria, or being discharged too early and investigators didn’t have control over the collected data since this study utilizes secondary data. In addition, we were unable to incorporate statistical methodologies which account for small sample size.

## Conclusions

Not only recovery and death rate but also the other outcome indicators in this study showed that there were in the minimum standard set of sphere project values/international standards. Increased recovery rate and reduced mortality rates among 6–59 months children in the study units were observed. Age group, folic acid supplementation and HIV status were predictors for recovery. Health facilities should strengthen folic acid supplementation and screening of HIV infection as early as possible at each service area.

## Abbreviations

AHR: Adjusted Hazard Rate
DALYs: Disability Adjusted Life Years
GDP: Gross domestic product
HIV: Human immunodeficiency virus
MUAC: Mid upper arm circumference
SAM: Severe acute malnutrition
SPSS: Statistical package for social sciences
WFH: Weight for height
WFL: Weight for length
